# Ten years of MRSA surveillance in Switzerland: similarities and differences with Europe

**DOI:** 10.1186/2047-2994-4-S1-O8

**Published:** 2015-06-16

**Authors:** F Olearo, W Albrich, S Harbarth, A Kronenberg

**Affiliations:** 1 SPCI, Geneva Univ. Hospital, Geneva, Switzerland; 2 Infect. Dis. Division, Cantonal Hospital St. Gallen, St. Gallen, Switzerland; 3 Dep. of Infect. Dis., Univ. Hospital Bern, Bern, Switzerland

## Introduction

The global epidemiology of methicillin-resistant *Staphylococcus aureus* (MRSA) is heterogeneous.

## Objectives

The objective of this study was to evaluate the epidemiology of MRSA in Switzerland over a 10-year period.

## Methods

We conducted a descriptive analysis of individual patient-level and aggregate MRSA data from the ANRESIS from 2004 to 2013.We also performed a time series analysis to characterize trends of MRSA and non-multidrug-resistant MRSA (Nm-MRSA, as a potential marker for community-associated MRSA, which was defined as being susceptible to at least three of the following agents: ciprofloxacin, clindamycin, tetracycline and trimethoprim-sulfamethoxazole (TMP/SMX) with stratification by Swiss regions,age-group and patient location.

## Results

Overall, 13,675 MRSA isolates were included. Although the proportion of MRSA among *S. aureus* (from 14% in 2004 to 10% in 2013 and the MRSA incidence decreased over time from 0.98 in 2004 to 0.58 per 1,000 discharged patients in 2013), an increasing trend of NmMRSA (+0.84% per quarter) was observed. Variation in the geographical distribution was noted, with a decrease in the proportion of MRSA among *S. aureus* in the Western region and Ticino (from 25% in 2004 to 16% in 2013 and from 20% to 15%) and stable and low prevalences (3-5%) in the Eastern and Central regions. We observed an increase in MRSA among *S. aureus* in outpatients (+0.044% per quarter) and a decrease in inpatients (-0.13% per quarter). Further analysis showed an increase in MRSA rates among *S. aureus* in younger age groups (2-15y,+0.11%per quarter) compared to decreasing MRSA rates in *S. aureus* for older age(>65 years –0.29% per quarter). Resistance to ciprofloxacin,clindamycin,gentamicin and erythromycin in MRSA strains decreased. Conversely, resistance to tetracycline, TMP/SMX, fusidic acid and rifampicin remained almost stationary and low over the study period.

## Conclusion

The proportion of MRSA among *S. aureus* in Switzerland decreased overall. The Ticino and West regions have moved from having a hyper-endemic MRSA prevalence comparable with neighboring countries,to lower levels of prevalence. MRSA appears to be an increasing problem in the younger population and in outpatients. The increased susceptibility to several antibiotic classes other than β-lactams suggests dissemination of strains, which have classically been reported as community-associated.

## Disclosure of interest

None declared.

